# Anti-Melanogenic Effects of Fractioned *Cynanchum atratum* by Regulation of cAMP/MITF Pathway in a UVB-Stimulated Mice Model

**DOI:** 10.3390/cells12101390

**Published:** 2023-05-14

**Authors:** Jing-Hua Wang, Seung-Ju Hwang, Sam-Keun Lee, Yujin Choi, Chang Kyu Byun, Chang-Gue Son

**Affiliations:** 1 Institute of Bioscience & Integrative Medicine, Daejeon University, 75, Daedeok-daero 176, Seo-gu, Daejeon 35235, Republic of Korea; wjhdon@dju.kr (J.-H.W.); bluesea9292@naver.com (S.-J.H.); 2 Department of Applied Chemistry, Daejeon University, Daejeon 34520, Republic of Korea; lsk236@dju.kr (S.-K.L.); byunck@dju.kr (C.K.B.); 3 Department of Internal Medicine, College of Korean Medicine, Se-Myung University, Jecheon-si 27136, Republic of Korea; chyj433@naver.com

**Keywords:** melanogenesis, *Cynanchum atratum*, UVB, melanin, tyrosinase, MITF, cAMP

## Abstract

Based on traditional pharmacological applications and partial in vitro data, *Cynanchum atratum* (CA) is proposed to act on skin whitening. However, its functional evaluation and underlying mechanisms have yet to be identified. This study aimed to examine the anti-melanogenesis activity of CA fraction B (CAFB) on UVB-induced skin hyperpigmentation. Forty C57BL/6j mice were exposed to UVB (100 mJ/cm^2^, five times/week) for eight weeks. After irradiation, CAFB was applied to the left ear once a day for 8 weeks (the right ear served as an internal control). The results showed that CAFB significantly reduced melanin production in the ear skin, as indicated by the gray value and Mexameter melanin index. In addition, CAFB treatment notably decreased melanin production in α-MSH-stimulated B16F10 melanocytes, along with a significant reduction in tyrosinase activity. Cellular cAMP (cyclic adenosine monophosphate), MITF (microphthalmia-associated transcription factor), and tyrosinase-related protein 1 (TRP1) were also noticeably downregulated by CAFB. In conclusion, CAFB is a promising ingredient for treating skin disorders caused by the overproduction of melanin and its underlying mechanisms involving the modulation of tyrosinase, mainly mediated by the regulation of the cAMP cascade and MITF pathway.

## 1. Introduction

The pathology of melanogenesis primarily involves melanin production process alterations, which can lead to various skin conditions or diseases, including hyperpigmentation, albinism, vitiligo, and melanoma [1]. Skin hyperpigmentation refers to the darkening of the skin caused by an increase in the production of melanin. Hyperpigmentation can occur due to a variety of factors, including exposure to UV radiation from the sun, hormonal changes, genetics, and skin trauma or inflammation. Therefore, skin whitening is the practice of bleaching the skin tone by reducing the concentration of melanin, which is the pigment that provides color to the skin [2]. The process of melanin synthesis commonly involves the conversion of tyrosine to dopaquinone, which can then be transformed into two different forms of melanin, including eumelanin (dark brown to black pigment) and pheomelanin (red to yellow pigment) [3]. The process is controlled by the key enzyme tyrosinase and its related proteins, such as tyrosinase-related proteins 1 and 2 (TRP-1 and TRP-2) [4]. These proteins are regulated by various factors, including microphthalmia-associated transcription factor (MITF), which binds to specific DNA sequences and activates the expression of genes involved in melanogenesis [5].

The promotion of skin whitening using cosmetics is currently of widespread global interest [6]. It is projected that the global market for skin-lightening products will surpass USD 800 billion in 2023, with an estimated annual growth rate of around 7% [7]. However, the use of certain skin-whitening products has been associated with a range of potential health risks, including poisoning, allergic dermatitis, kidney damage, and other adverse effects [8]. For example, hydroquinone and kojic acid, which are commonly used skin-whitening ingredients, have been linked to skin irritation, ochronosis, and allergic reactions [9]. Additionally, other ingredients, such as mercury and corticosteroids, can lead to poisoning, skin thinning, and permanent skin damage in case of long-term use. Consequently, there is a growing demand for safer, more natural, and effective skin whitening products globally, which has led to the development of products that employ plant-based ingredients. In response to these risks, natural herb-based skin-lightening products have gained increasing popularity [10]. 

*Cynanchum atratum* (CA), a perennial climbing plant of the family Apocynaceae, is a traditional herb medicine [11]. The root of CA has been widely used in oriental medicine for centuries to treat various ailments, such as swelling, postpartum vomiting, urinary infection, gonorrhea, nephritis, edema, bronchitis, and rheumatic arthralgia, for thousands of years [12]. Previous studies have shown that CA contains alkaloids and steroidal glycoside components [13] and exhibits multiple pharmacological activities, which have made it a valuable antidote, diuretic, and antipyretic [12]. Recently, extracts of CA have been increasingly utilized as a beneficial ingredient in cosmetics for skin whitening [14]. So far, more than a hundred ingredients in CA have been identified [15]. Cynanversicoside A, atratoglucoside A, cynanoside P1, and antofine are considered the potential active compounds from CA for inhibiting melanogenesis and inflammation [13,15,16,17]. However, several issues still need to be addressed, including safety, consistent efficacy, and potential mechanisms.

Therefore, the main objective of the current study was to evaluate the anti-melanogenesis property of the active fraction of *Cynanchum atratum* against UVB-induced skin hyperpigmentation and investigate its prospective molecular mechanisms.

## 2. Materials and Methods

### 2.1. Preparation of Cynanchum Atratum Fraction

**Figure 1:**
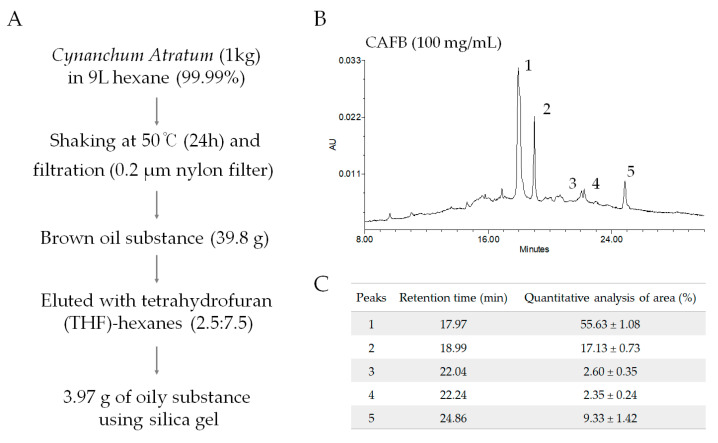
CAFB preparation and HPLC fingerprinting. (**A**) The procedure of *Cynanchum atratum* fraction B (CAFB) preparation. (**B**) HPLC fingerprinting of CAFB, (**C**) the retention time of the major peak, and relative quantitative analysis of the area.

The Korean pharmacopeia standard root of *Cynanchum atratum* (Latin name: Radix Cynanchi Atrati) was obtained from the Jeong-Seong Pharmaceutical Company (Daejeon, Republic of Korea), where it originates from Northeast China. The powder of *Cynanchum atratum* (1 kg) was mixed with 9000 mL of extra-pure hexane (CAS: 110-54-3, DUKSAN, Aansan, Republic of Korea) for 24 h at 50 ℃. The total hexane extract of *Cynanchum atratum* (HCA) was used as a control. In the present study, filtration of the hexane supernatants using a 0.2 μm nylon filter and removal of volatiles resulted in a brown oily substance (39.80 g), which was subjected to silica gel column chromatography. The fraction was removed and eluted with a mixture of tetrahydrofuran (THF)-hexanes (2.5:7.5) until no spot on a Thin Layer Chromatography (TLC) plate was detected by 254 UV light. The removed portion was vacuum-dried to acquire *Cynanchum atratum* fraction A (CAFA). The eluting THF collected the remaining compounds in silica gel, and the yellow oily portion (3.97 g, 0.39%) was obtained and considered *Cynanchum atratum* fraction B (CAFB) after removing volatiles under reduced pressure (Figure 1A). 

### 2.2. HPLC Fingerprinting Analysis

The HPLC instrument used was a Waters Alliance 2695 (Waters, Milford, CT, USA), which was equipped with a Waters 2996 photodiode array detector and autosampler. HPLC analysis was performed using a Luna C18(2) column (5 μm, 4.6 × 150 mm, Phenomenex, Torrance, CA, USA) with a mobile phase consisting of distilled water (A) and methanol (B) under the following gradient conditions: 0–10 min, 40–70% B (linear gradient); 10–15 min, 70–90% B (linear gradient); 15–20 min, 90–100% B (linear gradient); and 20–40 min, 100% B (isocratic). The photodiode array detector was set to 270 nm, the column temperature was 25 °C, and a flow rate of 1.0 mL/min was used. The injection volume was 10 μL (100 mg/mL). A total of five major peaks were detected at retention times of A (17.97 min), B (18.99 min), C (22.04 min), D (22.24 min), and E (24.86 min) at 270 nm (Figure 1B). Quantitative analysis for each peak was conducted relatively (Figure 1C). 

### 2.3. In Vitro Experiments

#### 2.3.1. Cell Lines and Culture

B16F10 murine melanoma cells (CRL-6475™) were obtained from ATCC, while RAW 264.7 cells (KCLB-40071) were purchased from the Korean Cell Line Bank (Seoul, Republic of Korea). Both cell lines were maintained in DMEM (Dulbecco’s modified Eagle’s medium, WelGENE, Daegu, Republic of Korea), supplemented with 10% fetal bovine serum (FBS, WelGENE) and 1% antibiotics (penicillin and streptomycin) in a humidified incubator at 37 °C and 5% CO2. All cells were sub-cultured during the logarithmic growth phase for future in vitro studies, and cell numbers were counted using the CellDrop™ Automated Bright Field Cell Counter (DeNovix, Wilmington, DE, USA).

#### 2.3.2. Cell Viability Assay

The cytotoxicity of CAFA and CAFB in B16F10 cells was determined using EZ-cytox cell viability detection kit (EZ-1000, Daeillab, Seoul, Republic of Korea). The cells were seeded in a 96-well plate at a density of 3 × 10^3^ cells/well; after 12 h incubation, the various concentrations of CAFs (0, 0.01, 0.1, 1, and 2 μg/mL) were added to each well in triplicate for 72 h. At the endpoint, 10 μL of EZ-cytox reagent was added to each well and incubated for 2 h at 37 °C. The absorbance of the supernatant was determined using a spectrophotometer at 450 nm (Molecular Devices, Sunnyvale, CA, USA).

#### 2.3.3. Melanin Contents Determination in B16F10 Cell

Cellular melanin concentration was measured using the previous method. B16F10 cells were seeded in 6-well plates at a density of 4 × 10^4^ cells/well. After 24 h incubation, the different concentrations of CAFs and HCA (0, 0.1, 1, and 2 μg/mL) were treated, and then 200 nm α-melanocyte-stimulating hormones (α-MSH) were added and incubated for 72 h. Kojic acid (1 μg/mL) was used as a positive control. Eventually, the cell supernatant was carefully removed and washed by DPBS (WelGENE, Daegu, Republic of Korea). After that, the gross images of each well were photographed using an Olympus IX71 microscope with a DP74 camera. Then, the attached cells were dissolved in 1 N sodium hydroxide (100 μL, NaOH, Sigma-Aldrich, St. Louis, MO, USA) at 95 °C for 1 h. The melanin content was determined according to the 450 nm absorbance of cell lysate using a spectrophotometer. 

#### 2.3.4. Tyrosinase Activity Assay in B16F10 Cell

The attached B16F10 cells from the same experiment as above were isolated using 200 μL lysis buffer containing 1% Triton X-100, 150 mM sodium chloride, 50 mM HEPES (pH 7.5), and 5 mM EDTA. After mixing with 2 mM L-Dopa and cell lysate, the mixture was incubated at 37 °C for 30 min. The mushroom tyrosinase (0 to 100 U) was used to obtain a standard curve for comparison. Finally, the absorbance at 450 nm was measured using a spectrophotometer. 

#### 2.3.5. Cyclic Adenosine Monophosphate (cAMP) Assay in B16F10 Cell

B16F10 Cell lysate cAMP was determined using a cAMP assay ELISA kit (Parameter^TM^ Mouse/rat cAMP Assay Kit, KGE 012B; R&D system; Minneapolis, MN, USA) according to the instruction from manufacturers.

#### 2.3.6. Real-Time PCR in B16F10 Cell 

The total RNA from the B16F10 cells was isolated using QIAzol Lysis Reagent (cat. No. 79306, Qiagen, Hilden, German). After the purity and integrity evaluation of RNA, cDNA synthesis was performed by a High-Capacity cDNA Reverse Transcription Kit (Thermo Fisher Scientific, San Jose, CA, USA). The equal cDNA, together with the PCR mix and corresponding primers (Table S1), were used for PCR amplification using a Rotor-Gene Q (Qiagen, Hilden, Germany). The β-actin gene was used as a housekeeping gene. The relative gene expression was represented by 2^−ΔCt^ and normalized by β-actin.

#### 2.3.7. Western Blot in B16F10 Cells 

B16F10 cells, which were from the same experiment for the melanin test, were isolated using a pro-prep^TM^ protein extraction solution (Intron Biotechnology Inc., Seongnam, Republic of Korea). The total protein concentrations of supernatant were determined by a BCA kit (Thermo Scientific, San Jose, CA, USA). The identical concentrations of protein samples were separated in 10% polyacrylamide gel electrophoresis and transferred to polyvinylidene fluoride (PVDF) membranes using the Mini-PROTEAN Tetra Cell System (BioRad, Hercules, CA, USA). The membranes were blocked using 3% bovine serum albumin (BSA), washed with Tween 20 thrice, and incubated with a primary antibody overnight at 4 °C (Table S2). Then, the membrane was incubated with horseradish peroxidase-conjugated IgG for 2 h. An advanced enhanced chemiluminescence (ECL) reagent (Thermo Fisher Scientific, San Jose, CA, USA) was used to screen the target protein. The final band on the membrane was detected by a FUSION Solo Image system (Vilber Lourmat, Marne-la-Vallée, France) and analyzed using ImageJ (1.52a, National Institutes of Health, Bethesda, MD, USA).

### 2.4. In Vivo Experiments

#### 2.4.1. Experimental Animal Design

Forty-six six-week-old female C57BL/6j mice were obtained from Daehan Biolink (Gyeonggi-do, Republic of Korea). After 10 days of environment adaption at 22 ± 2 °C and 50% ± 10% humidity with chow diet ad libitum, six animals were assigned to the normal group, while the other animals were divided randomly into five groups of eight mice each. Except for the normal group, forty C57BL/6j mice were exposed to UVB for 1 h per day (100 mJ/cm^2^, 5 times/week) for 8 weeks. CAFB (0.25%, 0.5%, and 1% dissolved in pure Vaseline) was applied to the left ear after exposure to UVB, while the right ear served as the internal control. At the end of the experimental day, the ear melanin contents were quantitatively evaluated by the Mexameter system (MX 18; Courage and Khazaka Electronic GmbH, Cologne, Germany) according to the Mexameter melanin index (MMI). The gray value of each ear photo was also analyzed using ImageJ (1.52a, National Institutes of Health, Bethesda, MD, USA). Then, the whole blood was collected from the abdominal aorta under isoflurane inhalational anesthesia (Hana Pharm, Hwaseong, Republic of Korea). The complete blood count (CBC) was performed using an Exigo Eos Veterinary hematology analyzer (Boule Medical AB, Spanga, Sweden). The ear tissues were removed and stored at −70 °C for the next analysis.

The animal study was in compliance with the Guide for Care and Use of Laboratory Animals (National Institutes of Health, 1996). The experimental design and protocol were approved by the Daejeon University Institutional Animal Care and Use Committee (approval No.: DJUARB-2022-016).

#### 2.4.2. Serum Biochemistry Measurement

After blood collection and 1 h clotting, blood was centrifuged at 3000× *g* for 15 min to separate the serum. Serum aspartate transaminase (AST), alanine transaminase (ALT), lactate dehydrogenase (LDH), blood urea nitrogen (BUN), creatinine (CRE), and total bilirubin were determined with an Auto Chemistry Analyzer (Chiron, Emeryville, CA, USA).

#### 2.4.3. Ear Skin Histological Observation by Fontana–Masson Staining

Both sides of the ear skin were fixed in 10% of formalin solution (Sigma, St. Louis, MO, USA). After gradual dehydration, the tissues were immersed in melted paraffin for three hours at least. Finally, the tissues were embedded in a paraffin block by a Leica embedding station (EG 1160, Nussloch, Germany). The paraffin-embedded tissues were sectioned into 5 μm.

#### 2.4.4. The cAMP Assay in Ear Tissue 

The cAMP levels in the left ear tissue were measured using a cAMP assay ELISA kit (Parameter^TM^ Mouse/rat cAMP Assay Kit, KGE 012B; R&D system; Minneapolis, MN, USA), following the manufacturer’s instructions.

#### 2.4.5. Western Blot in Ear Tissue

The left ear tissues were homogenized with RIPA buffer (Abcam, Cambridge, UK). After 12,000× *g* for 20 min centrifugation, the total protein concentrations of supernatant were also determined by a BCA kit (Thermo Scientific Scientific, San Jose, CA, USA). The procedure for protein separation and membrane transfer was identical to that used for the in vitro section (refer to Section 2.3.7.). After that, the membranes were then blocked, washed, and incubated with primary and secondary antibodies, as shown in Table S2. The final band was photographed using a FUSION Solo Image system (Vilber Lourmat, Marne-la-Vallée, France) and analyzed using ImageJ (1.52a, National Institutes of Health, Bethesda, MD, USA).

#### 2.4.6. Total Antioxidant Capacity in Ear Tissue

The serum and ear tissue levels of reactive oxygen species (ROS) were measured according to a previously described method [18]. To assess ROS levels in serum and ear tissue samples, 5 μL of each sample was mixed with 140 μL of sodium acetate buffer (0.1 M) and incubated at room temperature. Then, a rapid reaction was initiated by adding 5 μL of N, N-diethyl-para-phenylenediamine (DEPPD, 10 mM) and 95 μL of a ferrous sulfate mixture (4.37 μM). After a 1 min incubation, the ROS level was detected at 505 nm, with a standard curve generated using hydrogen peroxide (H_2_O_2_).

To determine total antioxidant capacity (TAC) in the left ear, a previously described protocol was followed [19]. Briefly, 10 μL of ear tissue sample homogenate was mixed with 90 μL of phosphate-buffered saline (PBS, 10 mM), 50 μL of myoglobin solution (18 μM), and 20 μL of 2,2′-azino-bis (3-ethylbenzthiazoline-6-sulfonic acid) diammonium salt (ABTS) solution (3 mM) and incubated for 3 min. The absorbance was then determined at 600 nm using a spectrophotometer after a 5 min incubation with 20 μL of 30% H2O2, with a gallic acid solution used as a standard.

### 2.5. Statistical Analysis

All the numerical results were indicated as the Mean ± Standard Deviation (SD) (*n* = 3 in vitro; *n* ≥ 6 in vivo) by SPSS software (19.0 version, Chicago, IL, USA). In the present study, a statistically significant difference was evaluated by one-way analysis of variance (ANOVA) followed by the least significant difference (LSD) post hoc test. A value of *p* less than 0.05 was considered statistically significant.

## 3. Results

### 3.1. CAFB Attenuated MSH-Stimulated Melanin in B16F10 Cells

**Figure 2:**
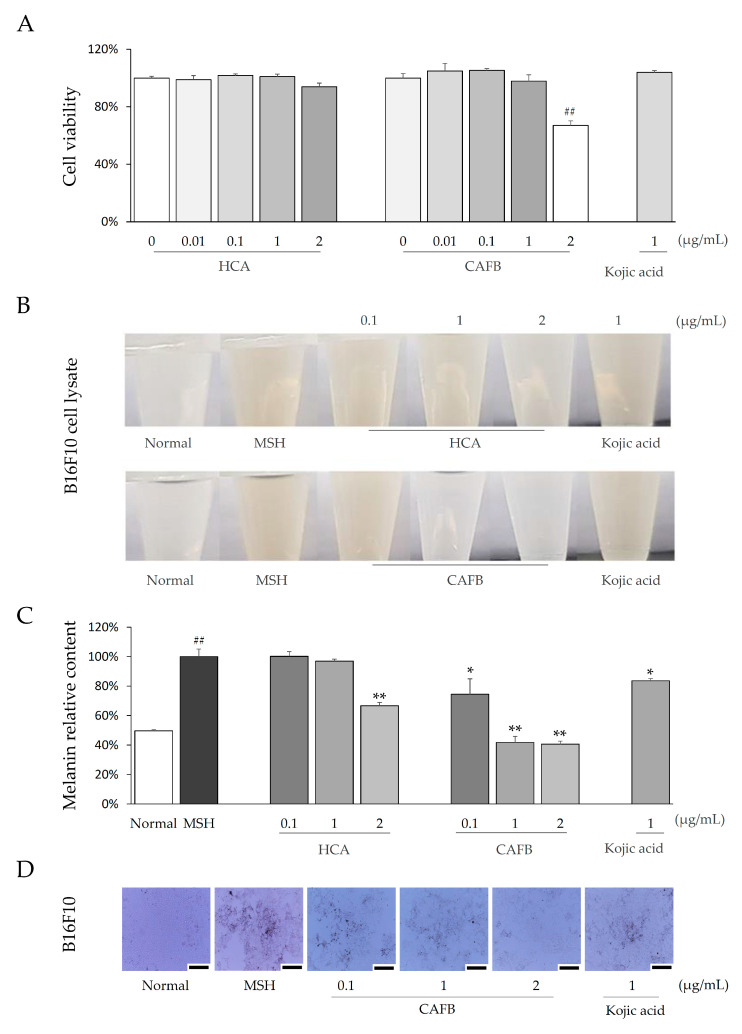
Melanin inhibition by CAFB in B16F10 cells. (**A**) Cell viability of HCA, CAFB, and Kojic acid in B16F10 was determined using an EZ-cytox cell viability detection kit. (**B**) The cell lysate of B16F10 was photographed. (**C**) The relative melanin contents were analyzed according to the OD value of B16F10 cell lysate. (**D**) The morphological change of B16F10 cells was photographed by the Olympus inverted fluorescent microscope (IX71, Tokyo, Japan) and digital camera (DP70, Tokyo, Japan). The length of the scale bars denotes 200 μm; ^##^ *p* < 0.01, as compared to the normal; * *p* < 0.05, ** *p* < 0.01, as compared to the MSH.

In B16F10 cells, no obvious cytotoxicity was observed from CAFB and HCA until a concentration of 1 μg/mL (Figure 2A). As expected, α-MSH treatment remarkably elevated melanin content in B16F10 cells, while CAFB (but not HCA and CAFA) significantly reduced melanin content even at a non-toxic concentration of 1 μg/mL. (*p* < 0.01, Figure 2B,C, Figure S1). The B16F10 cell morphology was also directly observed in keeping with the effect of melanin inhibition (Figure 2D). 

### 3.2. CAFB Regulates Tyrosinase Activity and Related Signaling in B16F10 Cells

**Figure 3:**
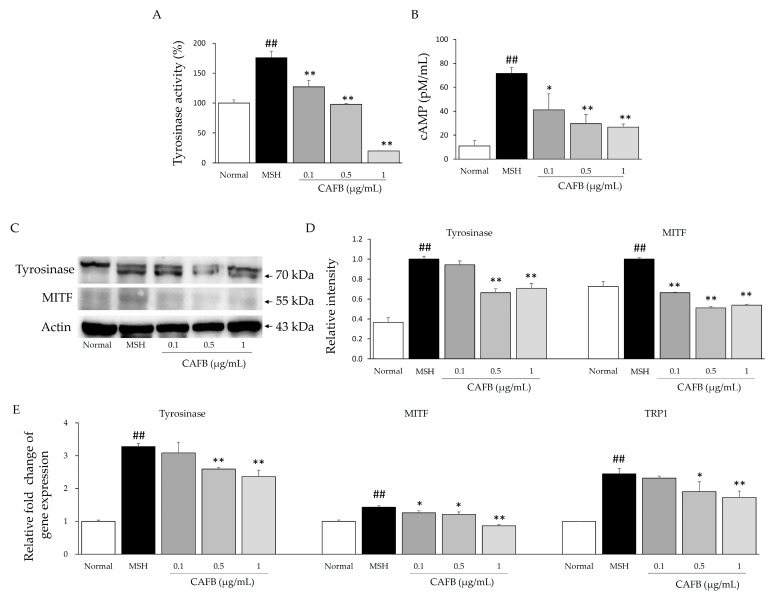
Potential anti-melanogenic mechanisms of CAFB in vitro. (**A**) Tyrosinase activity and (**B**) cAMP content in B16F10 cells was determined. Both (**C**,**D**) Western blot and (**E**) real-time PCR were performed using a CAFB-treated B16F10 cell model. All of the detailed contents are shown in the Section 2. ^##^ *p* < 0.01 as compared to the normal; * *p* < 0.05, ** *p* < 0.01 as compared to the MSH.

In B16F10 cells, CAFB significantly reduced α-MSH-induced hyperactivity of tyrosinase supported at both the protein and gene levels (Figure 3, *p* < 0.01). Furthermore, CAFB markedly decreased the excessive level of cAMP in a concentration-dependent manner. CAFB also apparently reduced the expression of MITF both at the gene and protein levels (Figure 3, *p* < 0.01 or 0.05). The TRP1 gene, as a crucial role in the production of melanin, was also down-regulated by CAFB (*p* < 0.01 or 0.05). 

### 3.3. CAFB Ameliorated UVB-Stimulated Skin Hyperpigmentation in Mouse Ears

**Figure 4:**
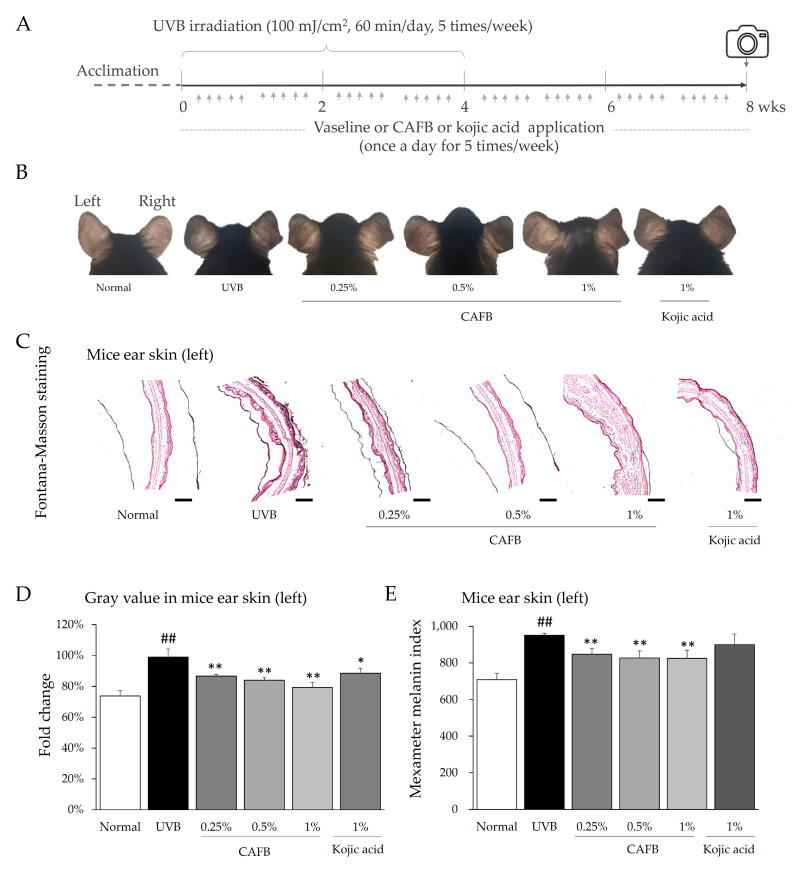
CAFB ameliorated UVB-stimulated skin hyperpigmentation in C57bl/6j mouse ear. (**A**) The experimental animal design. (**B**) Gross findings of mouse ear. (**C**) Melanin in the left ear tissue was confirmed by Fontana-Masson staining. The length of the scale bars denotes 200 μm. (**D**) Gray values of mouse ear skin were quantified by ImageJ software. (**E**) The Mexameter melanin index of mouse ear was measured using the Mexameter system, which is a specialized instrument for evaluating melanin levels. ^##^ *p* < 0.01 as compared to the normal; * *p* < 0.05, ** *p* < 0.01 as compared to the UVB.

Skin hyperpigmentation was visibly induced by UVB in the ears of mice as compared to non-irradiated mice (Figure 4B). CAFB led to a noticeable dose-dependent reduction in the gray value and MMI level of the skin on the left ear as compared to the controls (Figure 4D,E, *p* < 0.01). These findings were consistent with the gross observations (Figure 4B), resulting in skin brightening ranging from 13.2% to 20.7%. 

In addition, the histological observation using Fontana–Masson staining for ear skin also showed a mass of visualized melanin pigments in the cytoplasm of keratinocytes after UVB irradiation, whereas CAFB and kojic acid perceptibly reduced the accumulation of dark pigments (Figure 4C), whereas the same dose of CAFB exhibited a more extensive whitening effect than kojic acid (Figure 4C,D).

### 3.4. CAFB Inhibited the Melanin Synthesis via Downregulating the cAMP and MITF in Mouse Ears

**Figure 5:**
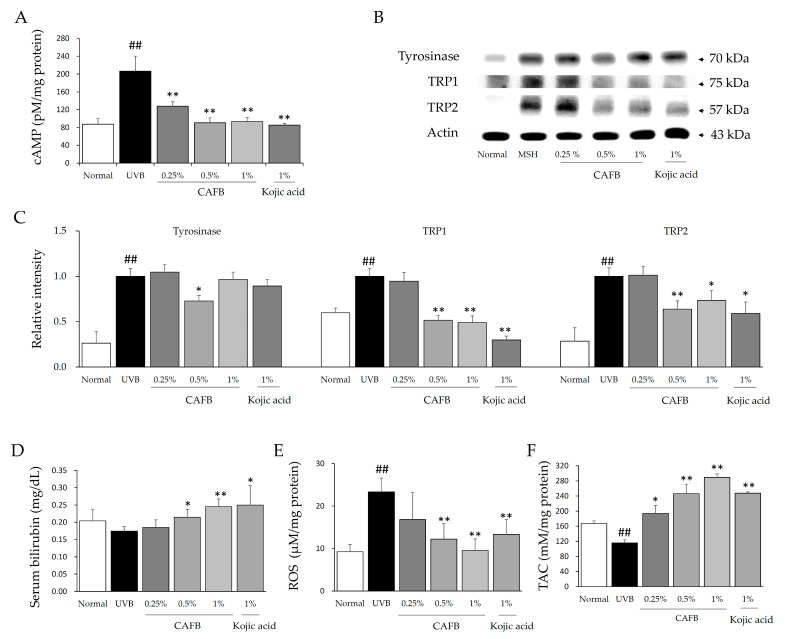
Potential anti-melanogenic mechanisms of CAFB in vivo. (**A**) cAMP content was determined by a cAMP Assay kit (R&D system; Minneapolis, MN, USA). (**B**,**C**) Left ear tissue was utilized for Western blot analysis of key proteins. (**D**) Serum bilirubin, as a potent endogenous antioxidant, was determined using an Auto Chemistry Analyzer (Chiron, Emeryville, CA, USA). (**E**,**F**) The levels of ROS and TAC in the left ear tissue were determined according to the corresponding methods. ^##^ *p* < 0.01 as compared to the normal; * *p* < 0.05, ** *p* < 0.01 as compared to the UVB.

Similar to the above in vitro results, CAFB application significantly decreased the levels of cAMP in the left ear tissue of mice (Figure 5A). CAFB also reduced the protein levels of tyrosinase, TRP1, and TRP2 in the left ear tissue of mice (Figure 5B,C), but this effect was not dose-dependent.

### 3.5. Anti-Oxidative Effect of CAFB in Mouse Ears

Exposure to UVB significantly increased the ROS level in ear tissue, whereas CAFB treatment notably reduced the ROS compared to UVB control (Figure 5E, *p* < 0.01). Serum bilirubin level in medium and high doses of the CAFB treatment group was significantly increased in a dose-dependent manner (Figure 5D, *p* < 0.05 or 0.01). Accordingly, UVB markedly reduced the TAC level in ear tissue, whereas CAFB treatment significantly recovered it in a dose-dependent manner (Figure 5F). High doses of CAFB (1%) treatment changed the ROS and TAC levels to a greater extent than the kojic acid (1%) treatment. Moreover, when compared to kojic acid, CAFB at a concentration of 50 μg/mL or higher showed a significantly greater capacity for scavenging DPPH free radicals (Figure S2).

### 3.6. CAFB Application Did Not Exhibit Discernible Negative Impacts on Skin and Body in Mice

Following an 8 week treatment of CAFB in combination with UVB irradiation, no discernible pathological alterations in ear skin, such as rash, redness, or blistering, were observed (Figure 4B). In addition, we observed no reductions in body weight or food intake as compared to UVB alone. We also did not find any signs of hepatotoxicity, nephrotoxicity, or hematological disorders based on typical serum markers (AST, ALT, LDH, BUN, and CRE) or CBC values (RBC, HGB, WBC, and PLT). However, only kojic acid in combination with UVB irradiation noticeably elevated the WBC count as compared to the normal group (*p* < 0.05, Table S3).

## 4. Discussion

As is known, when the skin is exposed to ultraviolet (UV) radiation from the sun, melanocytes are activated to produce more melanin to protect the skin from further UV-induced damage [20]. However, overexposure to UV radiation can lead to excessive production of melanin, resulting in hyperpigmentation of the skin [21]. In detail, UV radiation can stimulate melanocytes to secrete α-MSH, which is a hormone that contributes to skin darkening by increasing melanin production [22]. α-MSH binds to the MC1R receptor, which is predominantly expressed on melanocytes, simulating them to produce additional melanin [23]. The results from the α-MSH-induced B16F10 cell model indicated that CAFB, rather than CAFA, apparently attenuated the α-MSH-induced excessive melanin compared to α-MSH alone. Even though CAFB is derived from HCA, it was shown to be more effective in inhibiting melanogenesis compared to the same concentration of HCA and kojic acid. Taken together with the morphological findings of cells, these results clearly demonstrated that CAFB, as the active fraction of HCA, has a stronger potential anti-melanogenic activity than kojic acid, at least in vitro. Meanwhile, our in vitro data also showed that CAFB potentially exerts anti-inflammatory properties (Figure S3).

Tyrosinase is an enzyme that converts the amino acid L-tyrosine into dopaquinone within melanocytes [24]. After cyclization and oxidation, dopaquinone can form serval important intermediates, such as dopachrome and dopaquinone-2-carboxylic acid [25]. These intermediates are then further modified by enzymes called tyrosinase-related protein 1 (TRP1) and tyrosinase-related protein 2 (TRP2), which eventually lead to the formation of melanin [26,27]. Thus, tyrosinase plays a critical role in the process of melanin synthesis. Regulating the activity of tyrosinase and its associated enzymes is a crucial approach to decreasing melanin production [28]. Accordingly, downregulating tyrosinase is considered the most effective way to achieve skin whitening [29]. In our cell model, the activity of tyrosinase was inhibited dose-dependently by CAFB, as evidenced by all gene, protein, and enzymatic results. Additionally, the gene expression of TRP1 was downregulated by CAFB as well. Moreover, the reduction in TRP1 and TRP2 levels observed in the ear tissues of mice treated with CAFB indicates that its anti-melanogenic effect is primarily attributable to a decrease in the activity of these enzymes.

Next, we conducted an investigation into the corresponding mechanisms of melanin synthesis by assessing two critical signaling molecules [30,31], namely cAMP and MITF, in both cellular and animal models. Within melanocytes, cAMP acts as a secondary messenger and plays a crucial role in intracellular signal transduction triggered by α-MSH [32]. This cAMP signaling activates MITF, a transcription factor targeting the above molecules, including tyrosinase, TRP1, and TRP2, which act as master regulators of melanocyte differentiation and development [5,33]. In the current study, it was demonstrated that CAFB notably reduces the cAMP levels both in B16F10 cells and mouse ear tissues, which, consequently, led to decreased expression of MITF. We found that both CAFB and kojic acid had a comparable effect on cAMP levels in vivo. Multiple intracellular signaling pathways, including the PKA/CREB (protein kinase A/cAMP response element-binding protein) pathway [34], the MAPK/ERK (mitogen-activated protein kinase/extracellular-signal-regulated kinase) cascade [35], and the PI3K/Akt/GSK-3β (phosphoinositide 3 kinase/tyrosine kinase/glycogen synthase kinase 3β) pathway [36], are known to link cAMP to MITF activity. However, we could not ascertain the exact intracellular intermediate pathway or the specific active compounds involved in the present study.

In addition, UVB radiation, a major environmental stressor, can penetrate the skin and generate excessive ROS or other free radicals, which can activate signaling pathways that stimulate melanin synthesis [37]. On the other hand, UVB-induced oxidative stress causes damage to melanocytes, leading to increased transfer to keratinocytes, which can also contribute to skin hyperpigmentation [38]. Therefore, antioxidants, such as ascorbic acid and kojic acid, are commonly used as skin-whitening ingredients in cosmetics due to their ability to reduce oxidative stress [39,40]. In the present study, CAFB displayed superior DPPH free radical scavenging capacity compared to kojic acid. Bilirubin has been shown to have prominent endogenous antioxidant properties, including scavenging free radicals and protective properties against oxidative damage to DNA, which can help to protect cells suffering oxidative stress [41]. In line with the in vitro results, CAFB significantly increased the total bilirubin levels to neutralize ROS and enhance the total antioxidant capacity in mouse ear tissue. Consequently, we propose that CAFB exerts its anti-hyperpigmentation effect in the skin partially through the direct enhancement of antioxidant capacity. 

It is worth mentioning that the use of skin-whitening products has been linked to several adverse effects, including skin rash and allergic reactions [42]. Some products contain hydroquinone, which the International Agency for Research on Cancer (IARC) has classified as a possible human carcinogen, increasing the risk of cancer [42]. Some detrimental ingredients or improper usage can potentially cause damage to the organs and blood as well [43]. In our animal-based application of CAFB, the skin on the ear did not show any noticeable surface abnormalities along with no change in behaviors and any blood parameters, which at least preliminary indicates the safety of CAFB for external use. 

## 5. Conclusions

In summary, the active fraction of *Cynanchum atratum* effectively promotes skin whitening by reducing melanin production. The potential mechanisms include tyrosinase inhibition through restraining cAMP cascade and MITF pathway, as well as augmenting antioxidant capacity to eliminate excessive oxidative stress. Hence, these findings will be a valuable contribution to the development of functional agents for skin issues.

## Data Availability

The data that support the findings of this study are available within the article.

## Supplementary Materials

The following supporting information can be downloaded at: https://www.mdpi.com/article/10.3390/cells12101390/s1, Figure S1: CAFA did not inhibit the melanin synthesis; Figure S2: DPPH assay; Figure S3: Nitric oxide assay; Table S1: qPCR primer information; Table S2: Western blot antibodies information; Table S3: Body mass, food intake, serum biochemistry, and complete blood count [44,45].
