# Supplementary material for: Anti-Melanogenic Effects of Fractioned Cynanchum atratum by Regulation of cAMP/MITF Pathway in a UVB-Stimulated Mice Model
Source: Cells. 2023 May 14;12(10):1390. doi: 10.3390/cells12101390 (PMC10216695; doi:10.3390/cells12101390)
Supplement: Supplementary file 1 [file cells-12-01390-s001.zip › cells-2347312-supplementary.pdf]

## Supplementary methods

*1. DPPH scavenging assay*

The free radical scavenging activity of CAFB was tested using the DPPH assay. Different concentrations of CAFB (0, 1, 10, 50, 100, 500, 1000 μg/mL, and 100 μL) were mixed with 100 μL of 0.3 mM ethanolic DPPH in a 96-well plate. After 15 minutes of incubation at room temperature, the absorbance was detected using a spectrophotometer at 517 nm (Molecular Devices, Sunnyvale, CA, USA). An identical concentration of kojic acid was used as a positive control for comparison. The inhibition rate (%) was calculated using the following equation:

$$\text{DPPH free radical inhibition rate (\%)} = 1 - (OD_{control} - OD_{sample})/OD_{control} \times 100\%$$

*2. Nitric oxide assay in RAW 264.7 cell*

RAW 264.7 cells were adjusted to $1 \times 10^5$/well in a 24-well plate. After 24 hours, different concentrations of CAFB (0, 0.1, 0.5, and 1 μg/mL) were added to the wells and incubated for 4 hours. Next, lipopolysaccharide (LPS) (0.2 μg/mL) was added to all wells except the normal control and incubated for 24 hours. Finally, the supernatant was collected and used to assess the nitric oxide content according to the previous protocol [S1].

S1. Wang, J.H.; Shin, J.W.; Son, J.Y.; Cho, J.H.; Son, C.G. Antifibrotic effects of CGX, a traditional herbal formula, and its mechanisms in rats. J Ethnopharmacol 2010, 127, 534-542, doi:10.1016/j.jep.2009.10.001.

Figure S1 CAFA did not inhibit the melanin synthesis

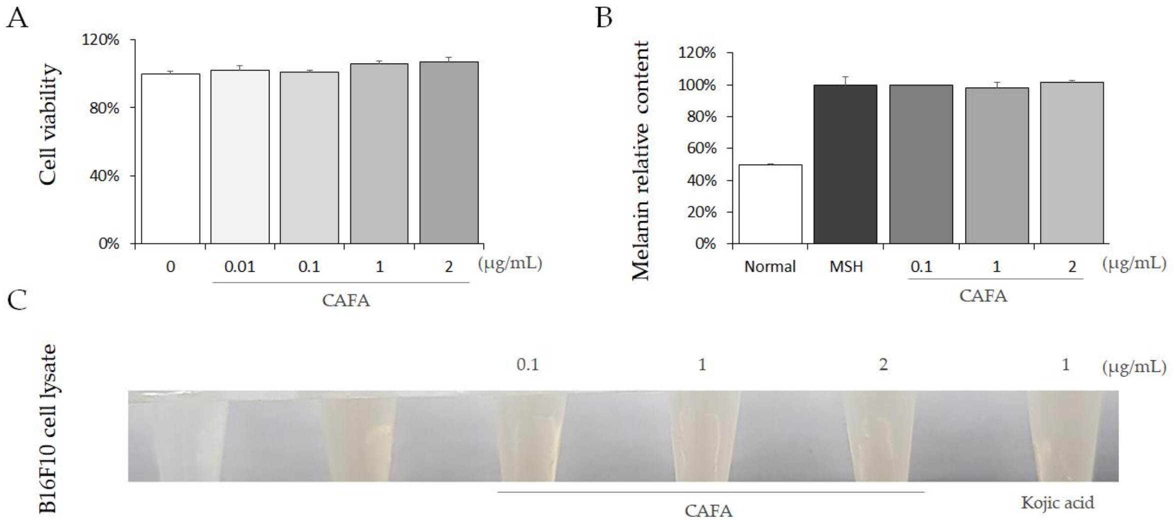


Figure S2 DPPH assay

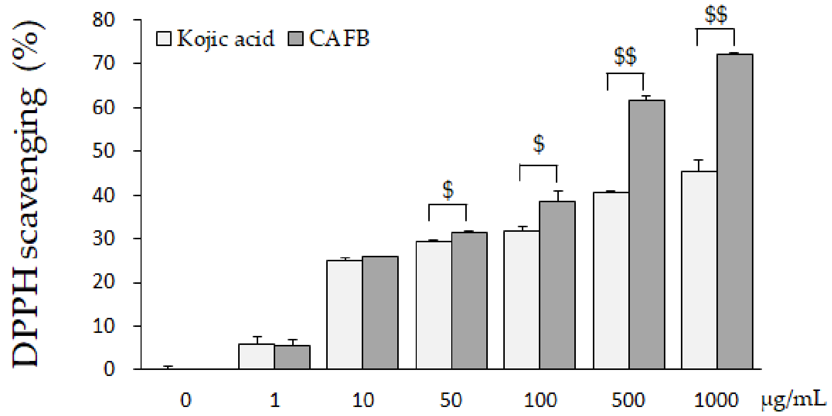

Figure S3 Nitric oxide assay

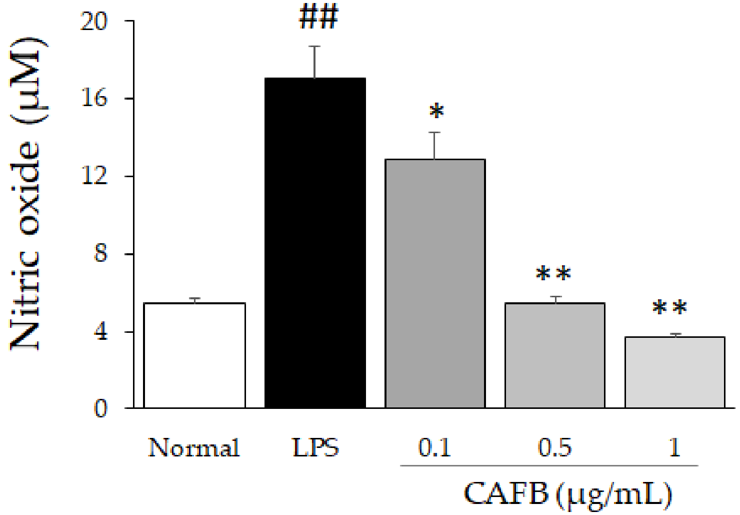

**Table S1.** qPCR primer information

| Gene name | Primer sequence | OAT | Ref. |
|---|---|---|---|
| GAPDH | 5′-CGT CCC GTA GAC AAA ATG GT-3′<br>5′-TTG ATG GCA ACA ATC TCC AC-3′ | 60°C | [S2] |
| TYR | 5′-TTG CCA CTT CAT GTC ATC ATA GAA TAT T-3′<br>5′-TTT ATC AAA GGT GTG ACT GCT ATA CAA AT-3′ | 60°C | [S2] |
| MiTF | 5′-CGC CTG ATC TGG TGA ATC G-3<br>5′-CCT GGC TGC AGT TCT CAA GAA-3′ | 60°C | [S2] |
| TRP1 | 5′-ATG CGG TCT TTG ACG AAT GG-3′<br>5′-CGT TTT CCA ACG GGA AGG T-3′ | 60°C | [S2] |

Abbreviations: OAT, optimized annealing temperature; ACTIN, β-actin; TYR, Tyrosinase; MiTF, Microphthalmia-associated transcriptian factor; TRP1, tyrosinase related protein 1

S2. Sato, K.; Takei, M.; Iyota, R.; Muraoka, Y.; Nagashima, M.; Yoshimura, Y. Indomethacin inhibits melanogenesis via down-regulation of Mitf mRNA transcription. Biosci Biotechnol Biochem 2017, 81, 2307-2313, doi:10.1080/09168451.2017.1394812.

**Table S2.** Western blot antibodies information

| Peptide/Protein target | Manufacturer | Catalog | Dilution ratio |
|---|---|---|---|
| Actin | Thermo Fisher scientific | MA 5-11869 | 1:1,000 |
| TYR | abcam | ab170905 | 1:1,000 |
| TRP1 | abcam | ab235447 | 1:1,000 |
| TRP2 | abcam | ab221144 | 1:1,000 |
| MiTF | abcam | ab12039 | 1:1,000 |
| Goat Anti-Rabbit IgG (HRP) | GeneTex | GTX213110-01 | 1:5,000 |
| Goat Anti-Mouse IgG (HRP) | GeneTex | GTX213111-01 | 1:5,000 |

Abbreviations: Actin, β-actin; TYR, Tyrosinase; MiTF, Microphthalmia-associated transcriptian factor; TRP1, tyrosinase related protein 1; TRP2, tyrosinase related protein 2; HRP, horseradish peroxidase; IgG, Immunoglobulin G.

**Table S3.** Body mass, food intake, serum biochemistry, and complete blood count.

| Contents | Normal | UVB | CAFB 0.25% | CAFB 0.5% | CAFB 1% | Kojic acid 1% |
|---|---|---|---|---|---|---|
| Body mass (g) | 23.0 ± 1.2 | 21.1 ± 1.5# | 20.9 ± 1.0 | 22.2 ± 1.0 | 21.3 ± 0.8 | 21.1 ± 1.2 |
| Food intake (g/mice/day) | 6.57 ± 0.13 | 5.40 ± 0.55 | 5.60 ± 0.89 | 5.87 ± 0.59 | 6.04 ± 0.87 | 5.45 ± 0.50 |
| AST (IU/L) | 41.5 ± 5.7 | 42.9 ± 5.1 | 42.2 ± 5.0 | 41.7 ± 5.1 | 45.8 ± 2.6 | 42.8 ± 4.6 |
| ALT(IU/L) | 19.1 ± 3.3 | 20.5 ± 3.2 | 21.7 ± 2.0 | 20.7 ± 1.8 | 23.5 ± 1.9 | 21.3 ± 1.8 |
| AST/ALT ratio | 2.19 ± 0.23 | 2.10 ± 0.14 | 1.94 ± 0.09 | 2.02 ± 0.26 | 1.97 ± 0.22 | 2.02 ± 0.30 |
| LDH (IU/L) | 189.3 ± 66.9 | 187.3 ± 80.8 | 145.3 ± 31.6 | 185.2 ± 31.6 | 183.9 ± 21.9 | 185.6 ± 59.4 |
| BUN (mg/dL) | 21.1 ± 2.2 | 20.9 ± 0.9 | 20.6 ± 2.1 | 21.6 ± 2.2 | 20.9 ± 2.5 | 21.0 ± 2.0 |
| CRE (mg/dL) | 0.33 ± 0.05 | 0.29 ± 0.06 | 0.32 ± 0.06 | 0.32 ± 0.10 | 0.32 ± 0.06 | 0.28 ± 0.06 |
| RBC ($10^9$/mL) | 7.8 ± 1.1 | 7.8 ± 0.5 | 8.0 ± 0.5 | 8.1 ± 0.6 | 7.9 ± 0.6 | 8.2 ± 0.5 |
| HGB (g/dL) | 12.4 ± 1.5 | 12.1 ± 0.6 | 12.5 ± 0.8 | 12.7 ± 1.0 | 12.6 ± 0.9 | 12.8 ± 0.7 |
| WBC ($10^6$/mL) | 9.2 ± 3.2 | 11.5 ± 3.4 | 10.5 ± 2.4 | 11.8 ± 2.5 | 11.8 ± 3.4 | 14.3 ± 2.1** |
| PLT ($10^6$/mL) | 608 ± 223 | 474 ± 207 | 539 ± 184 | 579 ± 247 | 492 ± 132 | 364 ± 102 |

AST, aspartate transaminase; ALT, alanine transferase; TBIL, total bilirubin; LDH, lactate dehydrogenase; BUN, blood urea nitrogen; CRE, creatinine; RBC, red blood cell; HGB, hemoglobin; WBC, white blood cell; PLT, platelets; UVB, ultraviolet B; CAFB, fraction B of *Cynanchum atratum*; $^{\#}p < 0.05$ as compared to the normal; $^{**}p < 0.01$ as compared to the UVB.
